# Postsurgical complications after robot-assisted transaxillary thyroidectomy: critical analysis of a large cohort of European patients

**DOI:** 10.1007/s13304-022-01244-2

**Published:** 2022-03-03

**Authors:** Leonardo Rossi, Valentina Buoni, Lorenzo Fregoli, Piermarco Papini, Andrea De Palma, Gabriele Materazzi

**Affiliations:** Department of Surgical, Medical and Molecular Pathology and Critical Area, University of Pisa, Pisa, Italy

**Keywords:** Robot-assisted trans-axillary thyroidectomy, Robotic surgery, Thyroid, Complications, Remote access

## Abstract

In the last decade, robot-assisted trans-axillary thyroidectomy has spread rapidly and has been proven to be a safe and effective procedure. However, several case series have reported new complications that have led to criticism regarding this approach. This study analyzed the incidence of complications in a large cohort of European patients. We enrolled all patients who underwent robot-assisted trans-axillary thyroidectomy from 2012 to 2020 at the University Hospital of Pisa Department of Endocrine Surgery. We analyzed complications and divided them into 2 groups. Group A included conventional complications, such as transient or permanent recurrent laryngeal nerve palsy, transient or permanent hypocalcemia, hemorrhage, and tracheal injury. Group B included unconventional complications, such as brachial plexus palsy, track seeding, seroma, great vessels injury, and skin flap perforation. There were 31 postsurgical complications (5.7%). Group A included 25 complications (4.6%): transient and permanent recurrent laryngeal nerve palsy occurred in 7 patients (1.3%) and in 1 (0.2%), respectively; transient and permanent hypocalcemia occurred in 9 patients (1.7%) and in 1 (0.2%), respectively. Postoperative bleeding occurred in 6 patients (1.1%) and tracheal injury in 1 (0.2%). Group B included 6 complications (1.1%): 1 patient with brachial plexus injury (0.2%), 1 with track seeding (0.2%), and 4 with seroma (0.7%). Robotic trans-axillary thyroidectomy is a safe approach with a risk of postoperative complications comparable to the conventional technique. Almost all complications after a novel introduction are anecdotal. With an accurate patient selection, high-volume institutions with experienced surgeons can perform this technique safely.

## Introduction

In the last 2 decades, endoscopic procedures for thyroid surgery with alternative access to the conventional Kocher incision have spread, leading to improvements in the fields of immediate postoperative pain and cosmetic results [1–3]. All of these are characterized by the classic limitations of endoscopic surgery, including 2-dimensional (2D) view, narrow surgical space, and rigid endoscopic instruments [4, 5].

The da Vinci robotic system (Intuitive Surgical, Sunnyvale, CA), which was approved by the United States Food and Drug Administration in 2006 [6], has been able to overcome some of these limitations. By means of the magnified vision of the surgical field, the 3D view, the tremor filtering system, and the endo-wrist technology along with the multi-articulations of the arms (7 degrees of freedom), the da Vinci robot allows wider and finer movements and reduces the interference between robotic arms and the camera [7].

In 2007, Chung and colleagues introduced robot-assisted trans-axillary thyroidectomy (RATT) using the da Vinci robotic system [8, 9]. This technique spread rapidly in Eastern countries, where thousands of procedures were performed in the following years. Its success in the Western world came more slowly as a result of various factors, including cultural and anthropometric differences [10].

At the beginning, concerns were raised about new complications associated with the RATT procedure. These include traction injury of the brachial nervous plexus [11], perforation or other thermic damage to the axillary skin flap, vascular lesions, formation of seromas in the chest wall, and re-implantation of thyroid tissue along the surgical track [12, 13]**.** However, data reported in the literature suggest a low incidence of these complications [14]**.** The incidences of classic postoperative complications with RATT, such as hypoparathyroidism, recurrent laryngeal nerve (RLN) palsy, and postoperative bleeding, are reported to be comparable to those of conventional technique [15].

Robotic thyroidectomy has therefore proved to be a safe technique in both postoperative complications and oncologic radicality in the treatment of thyroid carcinomas [16, 17]. However, large case series in European patients are still lacking in the literature, and the debate about its feasibility and efficacy for this cohort continues. The aim of this study was to analyze the incidence of complications, both conventional and of novel introduction, of RATT on a large series of European patients.

## Materials and methods

A prospectively designed database was used for data collection and analysis. We enrolled all patients who underwent RATT from February 2012 to December 2020 at the University Hospital of Pisa Department of Endocrine Surgery. All procedures were performed with the da Vinci robotic system, both the SI and the XI versions, using a single axillary incision and 3 robotic arms. Data regarding patient demographics, extent of surgery, preoperative diagnosis, thyroid volume and diameter of nodules, operating time, postoperative hospital length of stay, and postoperative complications were recorded. 

Selection criteria for RATT were a sonographically estimated thyroid volume up to 30 mL and a maximal sonographically determined nodule diameter up 60 mm for benign nodules and up to 30 mm for malignant nodules. Exclusion criteria were previous neck surgery, locally advanced cancer, lymph node metastasis at diagnosis, distant metastasis at diagnosis, severe thyroiditis, and Graves’ disease. The choice of hemi-thyroidectomy or total thyroidectomy was in concordance with the American Thyroid Association (ATA) guidelines at the time.

### Postoperative complications

We analyzed complications and divided them into 2 groups. Group A included conventional complications, which are defined as complications that could occur after standard open thyroidectomy, such as transient or permanent RLN palsy, transient or permanent hypocalcemia, hemorrhage, or tracheal injury. Group B included nonconventional complications, which are defined as complications of novel introduction mainly related to the remote access or to the position of the patient, such as brachial plexus palsy, surgical track seeding, seroma, great vessels injury, or skin flap perforation.

All patients underwent postoperative laryngoscopy to assess motility of the vocal cords. Permanent chordal paralysis was defined as such if it persisted more than 6 months after surgery. Hypoparathyroidism was diagnosed in patients who had a reduction of serum calcium below 8 mg/dL. It was defined permanent if it lasted more than 6 months after the operation and required supplemental calcium and vitamin D oral therapy. Bleeding was defined as a hemorrhage that could be treated by surgical revision or by conservative therapy. Tracheal perforation was diagnosed when a subcutaneous emphysema developed and was confirmed by instrumental imaging. Brachial plexus injuries were identified in patients who presented pain and/or movement deficit in the arm or shoulder ipsilateral to surgical access. Clinical deficits were confirmed electrophysiologically by abnormal nerve conduction (neurapraxia) [18].

## Results

Between February 2012 and December 2020, 541 patients underwent RATT at the University Hospital of Pisa Department of Endocrine Surgery, including 537 women (99.3%) and 4 men (0.7%). Mean age was 36.6 years (range 16–73 years), and mean body mass index (BMI) was 21.3 kg/m^2^ (range 18–25 kg/m^2^). Mean diameter of the nodules was 25 mm (range 5–60 mm), and mean thyroid volume was 18.4 mL (range 9–25 mL).

The surgical extent included 203 total thyroidectomies (37.5%), 336 lobectomies (62.1%), and 2 isthmusectomies (0.4%). Mean operative time was 95 min for total thyroidectomies, 79 min for lobectomies (taking into considerations 33 lobectomies in which a frozen section was performed), and 60 min for isthmusectomies. Conversion to standard cervicotomy was required in only 1 procedure (0.2%) due to locally advanced cancer infiltrating the internal jugular vein that escaped preoperative diagnosis (Table 1). Mean postoperative hospital length of stay was 2.4 days (range 1–11 days).

**Table 1:** Clinical and pathologic features of RATT patients

Features of patients	Total RATT operations (*n* = 541)
Age, mean (range), y	36.6 (16–73)
Sex, *n* (%)	
Males	4 (0.7)
Females	537 (99.3)
Body mass index, mean (range), kg/m^2^	21.3 (18–25)
Nodule size, mean (range), mm	25 (5–60)
Thyroid volume, mean (range), mL	18.4 (5–25)
Pathology, *n* (%)	
Papillary thyroid carcinoma	247 (45.7)
Follicular carcinoma	11 (2.0)
Undetermined nodule	115 (21.3)
Nodular goiter	126 (23.3)
Multinodular goiter	5 (0.9)
Plummer adenoma	2 (0.4)
Noninvasive follicular thyroid neoplasm with papillary-like nuclear features (NIFTP)	16 (2.9)
Normal thyroid tissue (completion thyroidectomy)	19 (3.5)
Operation extent, *n* (%)	
Total thyroidectomy	203 (37.5)
Hemithyroidectomy	336 (62.1)
Isthmusectomies	2 (0.4)
Operative time, mean (range), min	
Total	85 (35–325)
Working space	17 (10–53)
Docking	6 (4–25)
Console	62 (25–280)
Postoperative hospital stay, mean (range), d	2.4 (1–11)
Conversions to standard cervicotomy, *n* (%)	1 (0.2)

Overall, 31 postsurgical complications occurred (5.7%) (Table 2). Group A included 25 complications (4.6%). Transient RLN palsy occurred in 7 patients (1.3%) and spontaneously resolved without medical therapy or speech rehabilitation, and permanent RLN palsy was documented in 1 patient (0.2%). Transient hypocalcemia was reported in 9 patients (1.7%), who were treated with oral calcium and vitamin D therapy until resolution, and permanent hypocalcemia occurred in 1 patient (0.2%). Postoperative bleeding occurred in 6 patients (1.1%), among whom 2 (0.4%) required endoscopic surgical revision (using the same axillary surgical access) and 4 (0.7%) were treated conservatively by local compression and antibiotic coverage. Tracheal injury was documented in 1 patient (0.2%) 40 days after the operation and was successfully treated by means of curettage of the edges and fibrin glue application during bronchoscopy.

**Table 2:** Perioperative complications

Variable	No. (%)
Overall complications	31 (5.7)
Group A—conventional complications	25 (4.6)
Recurrent laryngeal nerve injury	8 (1.5)
Transient	7 (1.3)
Permanent	1 (0.2)
Hypocalcemia	10 (1.9)
Transient	9 (1.7)
Permanent	1 (0.2)
Hematoma postoperative bleeding	6 (1.1)
Reoperation	2 (0.4)
Conservative management	4 (0.7)
Tracheal injury	1 (0.2)
Group B—unconventional complications	6 (1.1)
Seroma	4 (0.7)
Great vessels injury	0
Brachial plexus injury	1 (0.2)
Chyle leakage	0
Axillary skin flap perforation	0
Track seeding	1 (0.2)

Group B included 6 complications (1.1%). A brachial plexus injury with hypofunctionality of a distal segment of a sensitive branch of ulnar nerve was documented in 1 patient (0.2%). Track seeding of a benign goiter was reported in 1 patient (0.2%), and the relapse was removed through an incision above the pectoralis major muscle under local anesthesia. Seroma occurred in 4 patients (0.7%) and was successfully treated by repeated needle aspiration at the outpatient clinic.

## Discussion

In the last 2 decades, RATT has become a widespread approach to treat thyroid diseases but is mostly limited to the Asian countries [10]. Indeed, Asian surgeons first proposed this new technique on the basis of patients’ request to avoid neck scars, most often young women with religious issues [19], whereas RATT is still being discussed in the West, and a few case series have reported data on a considerable cohort of patients [20–22].

Overall, higher BMI and different anthropometric characteristics, as well as larger size of nodules and goiters combined with the elevated inherent cost of robotic surgery and the need of training, hinder the diffusion of this approach in United States and Europe [23, 24]. Although RATT is considered feasible and oncologically safe and leads to superior cosmetic satisfaction, it currently plays a niche role in selected patients with appropriate pathology in high-volume institutions [23, 25, 26].

For a safe adoption of a novel technique, surgeons should ensure acceptable outcomes and minimize surgical complications [27]. Therefore, RATT requires surgeons to become familiar with a new view of the classic anatomy from the inferior and lateral direction and to face potentially nonconventional complications mainly related to the position of the patient or to the remote access to the thyroid lodge [27]. Indeed, some case series [12, 19, 20, 28] reported instances of neurologic deficits in the brachial plexus distribution, jugular vein or carotid artery injury, vagus nerve injury, trachea and esophagus injury, chyle leakage, and axillary flap perforation [24].

The overall incidence of postoperative complications in our case series was acceptable (5.7%). Most of the complications occurred in the conventional group (4.6%). The rates of transient and permanent RLN injury were 1.3% and 0.2%, respectively, which are comparable to those of conventional thyroidectomy [29]. The robotic platform provides an optimal visualization of the RLN by means of 3D-magnified view, enabling the rate of this complication to be kept within the low range. Ban et al. described similar results in a large case series of Asian patients, reporting transient RLN injury in 1.23% and permanent RLN injury in 0.23% [12]. Surgeons must pay attention to the active blade when using the ultrasonic shears due to the potential transfer of energy to the nearby RLN.

During the bilateral procedure, the contralateral RLN may be poorly visualized, especially at the beginning of the experience. Once the superior pedicle is dissected and the lobe is freed from the strap muscles, a gentle traction upward of the lobe and an accurate dissection enables surgeons to identify the contralateral RLN. Moreover, intraoperative nerve monitoring is available even for RATT, and this tool may guide surgeons in RLN identification or surgical strategy (2-step surgery in case of loss of signal). Although very rare, bilateral RLN injury is the most serious complication of bilateral thyroid surgery, leading to airway obstruction and potentially requiring tracheostomy. Notably, this complication did not occur in our patients.

We documented 9 patients (1.7%) with transient hypocalcemia that progressively resolved with oral calcium and vitamin D supplementation within 6 months, and permanent hypocalcemia in 1 patient (0.2%). Taking into consideration only bilateral procedures, these complications occurred respectively in 4.4% and 0.5% of cases. Again, we attribute these encouraging data to the 3D-magnified view and to the fine movements of the robotic system which allow a gentle dissection that avoids jeopardizing the parathyroid blood supply.

Notwithstanding, some debate persists in literature regarding the rate of transient hypocalcemia. A meta-analysis by Jackson et al. reported that RATT and open thyroidectomy (OT) have a similar complication rate, except for transient hypocalcaemia, which shows a higher risk after RATT [26]. However, a meta-analysis by Shen et al. reported that RATT and OT showed comparable complications rates even for hypocalcemia [15]. Similarly, in a meta-analysis comparing robotic thyroidectomy and OT for thyroid cancer, Pan et al. indicated comparable results between these 2 approaches [25].

Postoperative bleeding is a potentially life-threatening event in thyroid surgery due to the risk of airway obstruction and may require emergency surgical treatment. The incidence of postoperative hemorrhage ranges between 1.2 and 1.5% [29, 30] and occurred in 6 patients (1.1%) in our series. However, only 2 (0.4%) required revision surgery. In particular, both patients were treated by re-exploration via the axillary access. The middle thyroid vein was identified as the source of bleeding in 1 patient, whereas in the other patient, the origin of the hemorrhage was attributed to a muscular vessel. The other 4 patients (0.7%) were successfully managed by applying a compressive dressing along the surgical track. Several studies reported a comparable rate of postoperative hemorrhage between RATT and OT [10, 15, 26].

Our data suggest that dissection and hemostasis performed by the robotic system are safe. We believe that the effectiveness of the harmonic scalpel helps in obtaining this result, thanks to the absolute immobility and right pressure on the branches produced by the robot and not influenced by the surgeon. Moreover, the working space created to access the thyroid lodge plays a protective role, allowing the blood to evacuate in the vast subcutaneous pocket in the pre-pectoral region, breaking down the risk of airway compression [12, 21].

A serious and threatening complication after thyroid surgery is tracheal injury, which occurred in only 1 patient (0.2%) in our case series, an incidence comparable to that reported by Asian authors [12]. The patient in our series presented with a delayed tracheal rupture and was treated 40 days after surgery conservatively by means of fibrin glue during bronchoscopy. The delayed tracheal leakage was likely caused by a very small injury on the anterior tracheal surface due to an inadvertent touch with the active blade of the harmonic scalpel, which was responsible for a slow, progressive, ischemic damage [31].

Regarding nonconventional complications, we reported 1 traction injury of the ulnar nerve (0.2%) at the beginning of the experience. The patient presented with neurapraxia and underwent a neurologic examination and electromyography that documented a hypofunctionality of the sensory component of the ulnar nerve. After a couple of weeks of physiotherapy rehabilitation, the sensorial deficit almost resolved, although a small area of hyposensitivity of the fifth finger persists.

The brachial plexus injury usually occurs during the first 20 to 40 min of surgery and is probably caused by extreme extension of the arm position or extreme flap retraction [11]. A proper arm position with the supine patient’s ipsilateral arm extended at the shoulder, flexed at the elbow, and fixed to an elevated padded board is necessary to prevent such complication. Somatosensory-evoked potentials monitoring for ulnar, radial, and median nerves, if properly used, are suggested to further reduce the possibility of brachial plexus nerves injury [32].

Another unconventional complication is the track seeding along the surgical access. Iatrogenic implantation refers to the desquamation and dissemination of cells that develop into nodular recurrences. Some case reports have described this clinical condition for malignant and benign diseases [33–36]. In our case series, track seeding of a benign goiter occurred in 1 patient (0.2%), despite our routine use of an endobag to retrieve resected specimens. Our hypothesis is that cell exfoliation occurred as a result of repeated minor trauma inflicted by the rigid endoscopic instruments and the repeated contact between these instruments and the surgical track before the specimen was placed into the bag. In addition, the highly vascularized pectoralis major muscle provided a growth-promoting environment for the desquamated cells. The small nodule along the track was removed under local anesthesia and was determined to be macro-follicular thyroid tissue at histologic examination [13].

Albeit rarely documented after conventional thyroidectomy, RATT harbors a higher potential to lead to postoperative seroma formation due to the wide subcutaneous dissection from the axilla to the neck region. Although its incidence is reported elsewhere to occur in almost 2% of patients [12], we documented only 4 patients (0.7%) with seroma. All of the seromas in our case series were successfully treated by repeated aspiration at the outpatient clinic.

Other complications related to the access are the lesions of great vessels. An eventual injury of the external jugular vein can be managed by means of ligation without adverse effects. However, an immediate repair is necessary if an injury occurs to the carotid artery or internal jugular vein (IJV) during the access to the thyroid lodge. More complex carotid injuries require conversion to open surgery to have an adequate operative field and therefore greater control over the injured vessel. Bleeding from the IJV is more easily controllable, unless located in the caudal portion of the neck. In the event of a small tear of the IJV or of a branch, bleeding can be managed by means of direct robot-assisted suture or by application of an endoscopic clip by the assistant surgeon at the bedside [12].

Even chyle leakage can be considered an unconventional complication related to access, unless caused by a lymph nodes dissection (not performed with the robotic approach at our institution). However, all of these complications are anecdotal and never occurred in our case series.

Lastly, another complication introduced with the advent of RATT is the skin flap perforation when surgeons create the axillary pocket. Because the skin over the clavicle is very thin and has less fat, a meticulous dissection is necessary with special care not to perforate the skin or cause a subdermal burn by electrocautery [27]. Even this complication is not reported in our case series.

In light of our findings, we consider RATT as a safe procedure with comparable outcomes to conventional thyroidectomy in properly selected patients, and our data are in agreement with other published reports. Although RATT harbors the potential risk of technique-related complications of novel introduction, these are very rare. Of course, surgeons must keep in mind the possibility that these complications may occur and how to prevent them.

The main limitations of our study are the retrospective nature and the lack of a control group. Overall though, the outcomes that emerged despite these limitations demonstrate that the incidence of complications after RATT is very low even in a cohort of European patients, who are known to have a higher BMI, a larger thyroid volume, and a higher incidence of thyroiditis compared with Asian patients, all factors that are considered to increase the incidence of complications after robotic thyroid surgery [37]. To our knowledge, this is the largest case series of European patients described in literature to date, proving this technique as safe and effective even in this cohort.

Moreover, recognizing RATT as a valid option to treat thyroid diseases with outcomes comparable to conventional surgery, we are able to satisfy patients who reject a cervical scar, improving the cosmetic outcome that in some cultures represents a very important issue.

We want to underline that this is a case series of a high-volume institution in which RATT has been performed for several years and in which surgeons had gained experience in robotic and endocrine surgery. Indeed, surgeon’s experience plays a role of paramount importance in the management of a potential complication to ensure little or no long-term sequelae.

In conclusion, robotic trans-axillary thyroidectomy is a safe approach with a risk of postoperative complications comparable to the conventional technique. Procedure-related complications occur with a very low incidence and are almost all anecdotal. Therefore, high-volume institutions with experienced surgeons who have mastered robotic and endocrine surgery can perform this technique safely with optimal results. To obtain these outcomes, a proper patient selection is mandatory.
